# FACE-TO-FACE EXERCISES ARE NOT SUPERIOR TO TELE-REHABILITATION IN RECENT POST-OPERATIVE TOTAL HIP ARTHROPLASTY: A RANDOMIZED CLINICAL TRIAL

**DOI:** 10.1590/1413-785220243201e278202

**Published:** 2025-04-07

**Authors:** MATEUS DE COL BRAZEIRO, KAREN FERNANDA MUELLER, CAMILLA DA SILVA ROHDE, BRUNA DE MORAES LOPES, MARCELO FARIA SILVA

**Affiliations:** 1 Universidade Federal de Ciências da Saúde de Porto Alegre (UFCSPA), Departamento de Fisioterapia, Porto Alegre, RS, Brazil.; 2 Hospital de Clínicas de Porto Alegre (HCPA), Porto Alegre, RS, Brazil.

**Keywords:** Rehabilitation, Muscle strength, Osteoarthritis, Hip prosthesis, Reabilitação, Força Muscular, Osteoartrite, Prótese de Quadril

## Abstract

**Objective:**

compare a face-to-face exercise program (face-to-face group [FG]) to telerehabilitation (telerehabilitation group [TG]), in patients undergoing total hip arthroplasty (THA).

**Methodology:**

randomized clinical trial with 24 participants: 14 in the FG, which held weekly exercise sessions with face-to-face supervision in the clinic; and 10 in the TG, which performed exercises at home, with guidance from the booklet and weekly calls from the researchers. All participants underwent 6 weeks of intervention and were evaluated, by a blinded evaluator, in the pre- and post-intervention moments for: pain; kinesiophobia; functional; joint range of motion (ROM); and peak muscle torque (PT).

**Results:**

post-intervention only the TG (p = 0.018; d = 1,744) showed improvement in the results of the TUG, however both TG (p = 0.043; d = 1,876) and FG (p = 0.002; d = 1,854) showed improvement in the HHS results.

**Conclusion:**

telerehabilitation proved to be as effective as face-to-face rehabilitation in improving PT, ROM and functional capacity of patients in the initial stage of THA rehabilitation. It can be considered a low-cost and easy access alternative in this post-operative phase. ***Level of Evidence I, Randomized control trial.***

## RESUMO

**Objetivo:**

comparar um programa de exercícios face-to-face (GF) à telerreabilitação (GT), em pacientes submetidos à artroplastia total de quadril (ATQ).

**Metodologia:**

ensaio clínico randomizado com 24 participantes: 14 no GF, que realizou sessões semanais de exercícios com supervisão presencial na clínica; e 10 no GT, que realizou exercícios em casa, com orientação de cartilha e ligações semanais dos pesquisadores. Protocolo de 6 semanas de intervenção, foram avaliados pré e pós-intervenção para: dor (END); cinesiofobia (Escala de Tampa); capacidade funcional (HHS e TUG); amplitude de movimento articular e picos de torque muscular.

**Resultados:**

pós-intervenção somente o GT (p = 0.018; d = 1.744) apresentou melhora nos resultados do TUG, porém tanto GT (p = 0.043; d = 1.876) como GF (p = 0.002; d = 1.854) apresentaram melhora nos resultados do HHS. Houve aumento na ADM pós-intervenção, no GF, de 9 graus (p = 0.013; d = 1.028) na abdução e de 5 graus na extensão (p = 0.037; d = 0.949) do membro inferior operado (MIO), e no GT, de 18 graus (p = 0.028; d = 1.571) na flexão do MIO.

**Conclusão:**

a telerreabilitação mostraram-se tão eficaz quanto a reabilitação face-to-face na melhora dos PT, da ADM e da capacidade funcional de pacientes em fase inicial de reabilitação de ATQ. **Nível de evidência I, Estudo clínico randomizado.**

## INTRODUCTION

The number of THAs performed globally has been growing annually, with estimations pointing to a worldwide increase of 219% in THAs by 2046, indicating a higher cost for the health system. Despite the high quality of life indices (84-97%) reported, evidence suggests that individuals undergoing THA surgery may exhibit functional alterations, movement restrictions, and deficits in muscle strength postoperative for one or two years.^1^ ^)-(^ ^2^

Scientific literature shows great variation in intervention: home-based programs (orientations or booklets) ^(^ ^3^ ^)^ or exercises with face-to-face supervision; ^(^ ^3^ ^)-(^ ^6^ exercises with^6^ or without load; ^(^ ^4^ ^)-(^ ^7^ ^),(^ ^9^ frequency; ^(^ ^9^ protocol duration and postoperative period-ranging from right after hospital discharge. ^(^ ^5^ ^)-(^ ^8^

Some studies^8^ ^),(^ ^9^ ^),(^ ^10^ ^)^ investigated the delivery of home-based exercise protocols (supervised or not) performed immediate or late postoperative, which were able to improve muscle strength, functionality and gait speed in THA patients. ^(^ ^8^ ^)-(^ ^10^

Hence, this study compared the effects of a face-to-face supervised exercise protocol, performed in the clinical setting, with a home-based program (tele-rehabilitation), followed via remote monitoring, in patients undergoing THA surgery.

## METHODS

### Study design

A randomized clinical trial was conducted following the CONSORT Statement guidelines after approval by the Research Ethics Committee of the University (protocol 3.049.371) and was registered on ClinicalTrials (NCT3208829). Before starting the procedures, all participants red, agreed and signed the informed consent form. 

### Participants

Sample size calculation was based on a mean difference of 10.84Nm regarding the peak torque of the hip abductors^4^, assuming a standard deviation of 11 points for group 1 and 10, for group 2, an alpha level of 0.05 and a power of 80%. A minimum total of 56 patients was obtained, 28 in each group.

Both groups consisted of patients (men and women) over 45 years of age attended at three hospitals in Porto Alegre city, Brazil, for primary THA surgery (10-30 days postoperative). Exclusion criteria included individuals with postoperative complications (infections, deep vein thrombosis, prosthesis dislocation, periprosthetic fractures, and neural lesions), who underwent a surgical procedure on the lower limbs less than 6 months ago, with muscle injuries for less than 3 months in the lower limbs, who had cardiovascular diseases with the presence of disability (severe heart failure) and neurological diseases (stroke with sequelae, Parkinson’s disease, neurodegenerative diseases), and those who were already undergoing physiotherapy.

### Outcomes

Participants underwent two assessments: initial (pre-intervention), performed before randomization; and post-intervention, within 7 days after the end of the 6-week follow-up. Both evaluations were conducted by a blinded researcher. 

#### Pain and kinesiophobia

Pain intensity on the operated hip was measured by the Pain Numerical Scale (PNS) which consists of 11 points numbered from 0 to 10. Presence of kinesiophobia was assessed by the Tampa Kinesiophobia Scale translated, adapted and validated in Brazil.

#### Self-reported functional capacity

Self-reported functional capacity was measured by the Harris Hip Score (HHS) questionnaire^11^.

#### Objectively measured functional capacity

Objectively measured functional capacity was assessed through the Test Timed Up and Go (TUG), which has excellent validity and reliability. ^(^ ^11^ ^)-(^ ^12^

#### Range of Motion

Active range of motion (ROM) was assessed in both hips by a single evaluator using a fleximeter (model FL6010, Sanny, Brazil), which showed excellent intra-rater reliability in three measurements (ICC = 0.935-0.994; p < 0.05). Movements were evaluated in the following positions: hip flexion, hip extension and abduction, internal and external hip rotation. ^(^ ^13^ Participants performed each movement three times, and were interrupted if compensatory movements were observed in the pelvis or trunk. Analysis used the means of the three measurements of each movement.

#### Muscle strength

Muscle strength was evaluated by measuring the isometric peak torque (TP) in both lower limbs using a portable dynamometer (model HHD 01165, Laffayete, United States), which showed excellent intra-rater reliability over three measurements (ICC = 0.939-0.980; p < 0.05). For each muscle group, three maximal isometric contractions were performed lasting 5 seconds with a 30-second interval for rest. ^(^ ^14^ Assessment measured the strength of the following muscle groups: hip abductors, hip extensors, hip flexors, internal and external hip rotators. ^(^ ^14^ The means of the three measurements were used for analysis, and the PT values were normalized by body mass according to the equation PT = peak torque (Nm)/body mass (kg)x100.

Limb order was previously randomized using a mobile application (*Randomizers - www.random.org*).

### Randomization and allocation

After initial assessment, the participants were randomized into two physiotherapy treatment groups: Face-to-face (FG) and Tele-rehabilitation (TG). An assistant researcher, who was not involved in the data collection or the follow-up, was responsible for generating a numerical sequence using random.org and hide this information in opaque envelopes numbered in sequence. The assistant researcher responsible for the training protocols opened these envelopes at the end of the pre-intervention evaluation to allocate each individual to one of the two treatment groups.

### Protocols

#### Inpatient phase

All patients were operated by orthopedic hip surgeons, with over 10 years of experience, using a posterolateral approach and with early support release of the operated lower limb. During hospitalization, all patients received daily physiotherapy sessions.

#### Post-hospital discharge phase

Two 6-week protocols consisting of exercises to be performed bilaterally (operated and non-operated lower limbs), twice a week, respecting the care and restrictions recommended for THA postoperative were delivered.

Both protocols included the same exercises: bridge (elevation of the pelvis in supine position); knee and hip flexion in supine position; seated knee extension; sit down and stand up; orthostatic planting; knee flexion in orthostasis. Three sets of 12 repetitions were performed for each exercise.

After randomization, TG members received an illustrated booklet and general guidelines, as well as explanations and demonstrations of all the exercises to be performed at home, whereas the FG group performed the exercises under supervision of the researchers. TG was monitored through weekly calls from the researchers to resolve possible doubts.

### Statistical analysis

Data distribution was analyzed by the Shapiro-Wilk test. Despite the normal distribution found, we decided to adopt non-parametric tests due to sample size. Reliability and reproducibility of the peak torque measurements were analyzed by intraclass correlation coefficient (ICC). Comparison between groups used the Mann-Whitney test. Effect of the interventions was represented by relative variation, calculated using the equation: (Value_post_ - Value_pre_)/Value_pre_ x 100, in which Value_pre_ is the median of the pre-intervention measure and Value_post_ is the median of the post-intervention measure. Intragroup analysis used the Wilcoxon test and effect size (ES) through Cohen’s *d* [effect size = (M_post_ - M_pre_)/DP_Grouped_, in which M_post_ is the mean of the post-intervention measure, M_pre_ is the mean of the pre-intervention measure, and SD_Grouped_ is the pooled standard deviation of the pre- and post-intervention measures. Effects were considered to be: insignificant (*d* < 0.19); small (*d* = 0.20-0.49); medium (*d* = 0.50-0.79); large (*d* > 0.80). Descriptive analysis used central tendency (median) measures of the data and of the 25^th^ and 75^th^ percentiles. Significance level (a) was set at 5%. All statistical analyses were performed using the Statistical Package for Social Sciences (SPSS) for Windows (version 18.0).

## RESULTS

**Figure 1:**
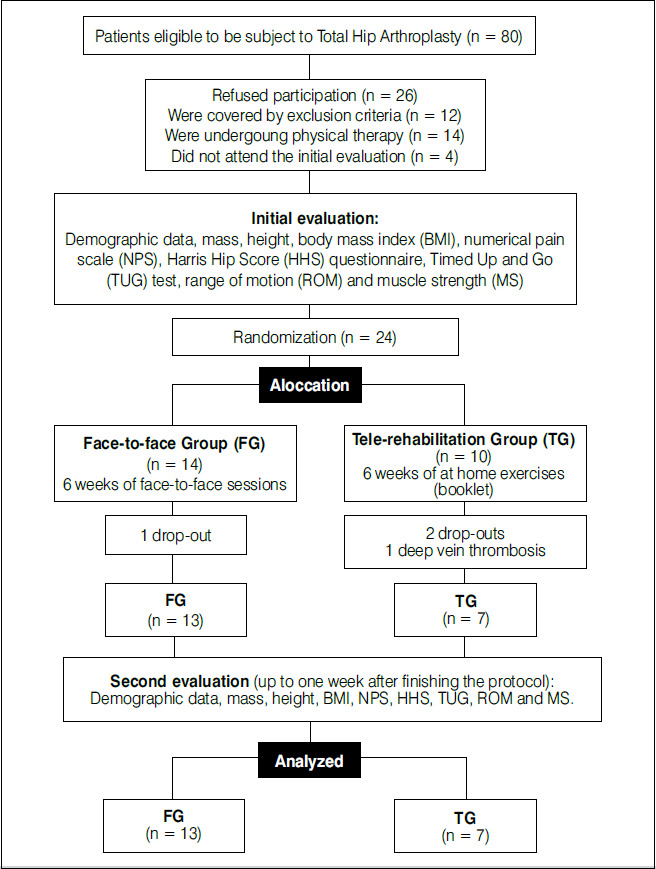
Sampling flow

From May 2018 to March 2020, we contacted 80 potential participants, of which 26 refused participation, 12 met exclusion criteria, 14 were already undergoing physical therapy, and 4 failed to attend initial assessment. Of the remaining patients, 24 met the inclusion criteria and were randomized into FG (n = 14) and TG (n = 10). Over the 6-week treatment, we had three dropouts (1 in the FG and 2 in the TG) and one patient (TG) developed deep vein thrombosis, and exercising is contraindicated, totaling four losses (Figure 1).

FG and TG showed no significant differences (*p*> 0.05) in the initial assessment regarding age, body mass index (BMI), disease duration, and postoperative time. Table 1 summarizes sample characterization.

**Table 1:** Sample characterization of the Tele-rehabilitation and Face-to-face groups.

Variables	Tele-rehabilitation Group (n = 7)	Face-to-face group (n = 13)	p
**Age** (years)	60.50 (47.00;69.00)	61.00 (59.00;67.75)	0.206
**BMI** (kg/m²)	28.80 (26.90;33.30)	30.10 (26.60;33.70)	0.968
Gender			
Male	2 (20%)	10 (71.42%)	
Female	8 (80%)	4 (28.58%)	
**Disease duration** (years)	5.00 (2.37;15.25)	6.00 (1.54;14.25)	0.837
**P.O. time** (days)	24.00 (19.00;30.00)	25.00 (19.00;30.00)	0.556
Type of prosthesis			
Cemented	3 (30%)	4 (28.60%)	
Non-cemented/hybrid	7 (70%)	10 (71.40%)	
Contralateral THA			
Yes	1 (10%)	3 (21.43%)	
No	9 (90%)	11 (78.57%)	
LL operated			
Dominant	4 (40%)	8 (57.14%)	
Non-dominant	6 (60%)	6 (42.86%)	

THA: total hip arthroplasty; BMI: body mass index; LL: lower limb; PO: postoperative. Data presented as median (P25;P75) or n (%).

Source: prepared by the authors.

FG and TG showed no significant differences (*p*> 0.05) in the initial assessment regarding age, body mass index (BMI), disease duration, and postoperative time. Table 1 summarizes sample characterization.

### Pain and Kinesiophobia

Both TG and FG exhibited low levels of pain according to the Numerical Pain Scale scores in pre and post-intervention evaluations, with no statistically significant differences between (pre-intervention *p* = 0.698; post-intervention *p*= 0.967) or intra-group (TG *p* = 0.443; FG *p*= 0.090). Regarding the kinesiophobia scores measured by the Tampa Scale, no significant differences were found when comparing pre- and post-intervention evaluations (TG *p* = 0.271; FG *p* = 0.461), or between groups (pre-intervention *p* = 0.905; post-intervention *p* = 0.266) (Table 2).

**Table 2:** Pain (numerical pain scale), kinesiophobia (Tampa Scale) and functional capacity (Harris Hip Score and Timed Up and Go) in the Tele-rehabilitation and Face-to-face groups.

		Tele-rehabilitation (n = 7)	Face-to-face (n = 13)	p	intergroup d
NPS	Pre	1.00 (2.00;5.00)	3.00 (1.00;6.00)	*0.698*	
	Post	1.00 (0.00;4.00)	1.00 (0.00;3.00)	*0.967*	*0.043*
	*intragroup p*	*0.443*	*0.090*		
	*intragroup d*	*0.477*	*0.662*		
Tampa	Pre	33.00 (31.00; 44.00)	37.00 (30.00;41.00)	*0.905*	
	Post	28.00 (27.00;41.00)	34.00 (30.00;38.00)	*0.266*	*0.315*
	*intragroup p*	*0.271*	*0.461*		
	*intragroup d*	*0.659*	*0.308*		
TUG(s)	Pre	44.40 (31.10;66.00)	24.10 (18.00;36.40)	0.036*	
	Post	17.50 (13.40;21.90)	16.30 (11.10;19.70)	*0.501*	*0.015*
	*intragroup p*	0.018*	*0.064*		
	*intragroup d*	*1.744*	*0.718*		
HHS	Pre	56.00 (40.80;61.70)	58.40 (49.60;68.80)	*0.285*	
	Post	81.50 (76.80;86.50)	78.20 (71.50;90.00)	*0.843*	*0.066*
	*intragroup p*	0.043*	0.002*		
	*intragroup d*	*1.876*	*1.854*		
				

NPS: Numerical pain scale; HHS: Harris Hip Score; TUG: Timed Up and Go. Data presented as median (P25;P75) or n (%); size of the effect represented by Cohen’s *d*; *p<0.05.

Source: prepared by the authors.

### Self-Reported and Objectively Measured Functional Capacity

Regarding self-reported functionality, patients had significantly higher HHS scores post-intervention compared with pre-intervention, with a large effect size both in the TG (*d* = 1.876) and FG (*d* = 1.854) groups (Table 2).

As for objectively measured functionality, we observed a significant difference (*p* = 0.036) between the groups pre-intervention, in which TG individuals needed more time to perform the TUG test. Both groups showed a reduction in the time taken to perform TUG in the post-intervention evaluation (TG = 26.9 seconds; FG = 7.8 seconds), but only the differences found for TG were significant (*p* = 0.018; *d* = 1.744). No significant differences were found (*p* = 0.501) between groups in the post-intervention evaluation (Table 2).

### Range of Motion

Participants showed a significant increase, with a large effect size, in the range of motion of the lower limbs post-intervention (Table 3). FG presented a statistically significant increase in abduction (p = 0.013; d = 1.028) and extension (p = 0.037; d = 0.949) in the operated lower limb, whereas TG showed a statistically significant increase in flexion (p = 0.028; d = 1.571) on the operated lower limb and in extension (p = 0.027; d = 1.298) on the non-operated lower limb (Table 3)

**Table 3:** Hip range of motion (ROM) pre- and post-intervention in the Tele-rehabilitation and Face-to-face groups, expressed in degrees.

		Operated Lower Limb			Non-Operated Lower Limb		
ROM		Tele-rehabilitation (n = 7)	Face-to-face (n = 13)	p	Tele-rehabilitation (n = 7)	Face-to-face (n = 13)	p
Abduction	Pre	13.00 (11.00;24.00)	15.00 (14.00;17.50)	*0.520*	25.00 (17.00;30.00)	22.00 (18.00;24.50)	*0.381*
	Post	23.00 (20.00;30.00)	24.00 (17.00;33.00)	*0.662*	29.00 (24.00;35.00)	25.00 (20.00;30.00)	*0.379*
	*intragroup p*	*0.075*	0.013*		*0.150*	*0.064*	
	*intragroup d*	*1.028*	*1.042*		*0.611*	*0.441*	
							
Extension	Pre	15.00 (15.00;19.00)	20.00 (15.00;24.00)	*0.166*	13.00 (12.00;24.00)	20.00 (15.00;29.00)	*0.102*
	Post	24.00 (14.00;30.00)	25.00 (19.50;29.00)	*0.577*	25.00 (20.00;30.00)	25.00 (18.00;27.50)	*0.905*
	*intragroup p*	*0.075*	0.037*		0.027*	*0.286*	
	*intragroup d*	*0.949*	*0.931*		*1.298*	*0.292*	
							
Flection	Pre	35.00 (24.00;43.00)	45.00 (34.00;51.00)	*0.218*	54.00 (35.00;79.00)	60.00 (43.00;63.50)	*0.905*
	Post	53.00 (49.00;58.00)	48.00 (40.00;62.50)	*0.605*	62.00 (51.00;66.00)	55.00 (39.50;65.50)	*0.427*
	*intragroup p*	0.028*	*0.136*		*0.672*	*1.000*	
	*intragroup d*	*1.571*	*0.723*		*0.252*	*0.025*	
							
Medial Rotation	Pre	15.00 (10.00;19.00)	14.00 (10.00;20.00)	*0.905*	20.00 (19.00;27.00)	16.00 (10.00;20.00)	*0.163*
	Post	15.00 (12.00;25.00)	15.00 (14.00;21.00)	*0.935*	23.00 (15.00;27.00)	22.00 (12.50;28.50)	*0.751*
	*intragroup p*	*0.612*	*0.381*		*0.752*	*0.123*	
	*intragroup d*	*0.411*	*0.276*		*0.161*	*0.022*	
							
Lateral Rotation	Pre	10.00 (6.00;11.00)	10.00 (5.50;14.50)	*0.874*	20.00 (15.00;30.00)	16.00 (12.50;21.50)	*0.190*
	Post	11.00 (10.00;12.00)	12.00 (10.00;15.00)	*0.354*	21.00 (15.00;25.00)	15.00 (11.00;18.50)	*0.095*
	*intragroup p*	*0.528*	*0.333*		*0.733*	*0.969*	
	*intragroup d*	*1.929*	*0.476*		*0.244*	*0.696*	
							

Data presented as median (P25;P75) or n (%); size of the effect represented by Cohen’s *d;* *p<0.05

Source: prepared by the authors.

### Muscle strength

TG and FG participants showed significant differences in torque peaks of the abductors and external rotators in the operated lower limb when comparing pre- and post-intervention evaluations. We observe an increase in TP of the abductors in TG (*p*= 0.028; *d* = 2.409) and FG (*p*= 0.023; *d*= 1.003), as well as an increase in TP of the rotators in TG (*p*= 0.018; *d*= 0.862) and FG (*p*= 0.016; *d*= 1.386) (Table 4).

**Table 4:** Pre- and post-intervention torque peaks in the Tele-rehabilitation and Face-to-face groups, expressed in Nm/kgx10.

		Operated Lower Limb			Non-Operated Lower Limb		
Torque Peaks		Tele-rehabilitation (n = 7)	Face-to-face (n = 13)	p	Tele-rehabilitation (n = 7)	Face-to-face (n = 13)	P
Abductors	Pre	58.46 (40.41;63.64)	85.66 (51.28;132.34)	*0.104*	123.65 (72.33;152.24)	114.20 (71.92;205.09)	*0.721*
	Post	128.04 (99.70;149.30)	148.26 (124.46;196.93)	*0.251*	148.49 (121.16;204.48)	159.07 (103.57;216.89)	*0.721*
	*intragroup p*	0.028*	0.023*		*0.091*	*0.345*	
	*intragroup d*	*2.409*	*1.003*		*0.772*	*0.206*	
							
Extensors	Pre	53.05 (47.24;115.01)	83.81 (65.53;156.66)	*0.166*	97.42 (57.48;135.51)	108.69 (88.28;187.45)	*0.219*
	Post	123.62 (52.08;157.09)	157.27 (75.37;184.61)	*0.501*	131.52 (60.07;196.35)	164.70 (94.47;230.05)	*0.322*
	*intragroup p*	*0.128*	*0.173*		*0.176*	*0.345*	
	*intragroup d*	*1.081*	*0.479*		*0.600*	*0.384*	
							
Flexors	Pre	86.14 (73.52;92.92)	173.16 (104.86;199.16)	0.008*	135.26 (105.82;204.62)	190.65 (148.55;231.71)	*0.088*
	Post	130.59 (114.59;169.65)	189.74 (148.86;231.92)	*0.036*	159.38 (99.47;202.45)	201.76 (132.47;263.53)	*0.251*
	*intragroup p*	*0.128*	*0.249*		*0.612*	*0.552*	
	*intragroup d*	*1.157*	*0.418*		*0.194*	*0.086*	
							
Internal Rotators	Pre	51.32 (38.43;63.48)	88.96 (52.79;109.97)	0.036*	78.77 (62.24;91.22)	100.73 (57.28;145.84)	*0.405*
	Post	74.24 (50.48;107.96)	71.91 (62.52;113.83)	*0.606*	102.04 (64.13;126.74)	103.56 (60.56;143.41)	*0.968*
	*intragroup p*	*0.310*	*0.917*		*0.499*	*0.861*	
	*intragroup d*	*0.861*	*0.031*		*0.055*	*0.029*	
							
External Rotators	Pre	41.71 (38.37;49.10)	43.26 (34.56;77.47)	*0.968*	98.40 (69.91;123.67)	108.56 (66.18;129.11)	*0.721*
	Post	80.17 (66.30;93.33)	99.78 (80.38;121.43)	*0.104*	113.02 (63.28;131.85)	123.93 (67.36;145.37)	*0.501*
	*intragroup p*	0.018*	0.016*		*0.866*	*0.279*	
	*intragroup d*	*0.862*	*1.386*		*0.445*	*0.292*	

Data presented as median (P25;P75) or n (%); size of the effect represented by Cohen’s *d;* *p<0.05

Source: prepared by the authors.

Table 5 presents the treatment effect (pre- and post-intervention difference) on pain, kinesiophobia, self-reported and objectively measured functional capacity, ROM and muscle strength. According to the relative variation results, the effect of the interventions was similar in both groups.

**Table 5:** Effect of the intervention on the Tele-rehabilitation and Face-to-face*,* represented by relative change (Δ).

	Δ Tele-rehabilitation (n = 7)	Δ Face-to-face (n = 13)	p
NPS	-80.00 (-95.00; 200.00)	-60.00 (-95.00; 00.00)	*1.000*
TAMPA	-25.80 (-36.40; 24.20)	-05.70 (-16.20; 19.40)	*0.405*
TUG (s)	-61.20 (-66.80; -59.00)	-33.70 (-64.20; -01.00)	*0.122*
HHS	50.00 (27.70; 112.00)	36.00 (21.80; 49.40)	*0.362*
Range of Motion			
Operated Lower Limb			
Abduction	66.70 (-19.20; 109.10)	60.00 (-07.70; 113.30)	*0.874*
Extension	60.00 (-18.80; 66.70)	25.00 (00.00; 66.70)	*0.721*
Flection	38.90 (14.00; 120.80)	25.00 (03.80; 41.30)	*0.322*
Medial Rotation	20.00 (-25.00; 133.30)	15.40 (-19.00; 60.00)	*1.000*
Lateral Rotation	10.00 (-09.10; 100.00)	00.00 (-14.30; 66.70)	*0.721*
Non-Operated Lower Limb			
Abduction	16.00 (-10.00; 50;00)	13.60 (07.10; 25.00)	*0.905*
Extension	25.00 (11.10; 92.30)	08.30 (00.00; 66.70)	*0.250*
Flection	20.40 (-16.50; 45.70)	00.00 (-21.40; 40.00)	*0.663*
Medial Rotation	26.30 (-34.30; 70.00)	35.00 (-06.30; 116.70)	*0.721*
Lateral Rotation	-20.00 (-30.00; 71.40)	06.70 (-15.00; 17.60)	*0.968*
Torque Peaks			
Operated Lower Limb			
Abduction	134.60 (38.10; 252.20)	80.90 (-14.70; 278.50)	*0.663*
Extension	105.30 (04.20; 239.10)	98.70 (-11.20; 134.20)	*0.552*
Flection	51.80 (28.70; 115.90)	16.30 (05.70; 70.80)	*0.362*
Medial Rotation	72.00 (38.80; 123.80)	151.90 (07.60; 226.70)	*0.606*
Lateral Rotation	01.20 (-01.90; 93.20)	15.70 (-45.30; 85.40)	*0.452*
Non-Operated Lower Limb			
Abduction	25.90 (04.90; 110.30)	19.10 (-13.30; 83.40)	*0.663*
Extension	35.00 (-16.70; 53.60)	23.90 (-12.70; 94.30)	*0.968*
Flection	18.90 (-16.20; 50.60)	02.10 (-17.80; 46.10)	*0.968*
Medial Rotation	-04.10 (-27.40; 25.30)	22.20 (-18.00; 51.30)	*0.606*
Lateral Rotation	13.30 (-29.70; 74.00)	-00.40 (-15.20; 21.80)	*0.843*

Data presented as median (P25;P75); p < 0.05

Source: prepared by the authors.

## DISCUSSION

After the 6-week application of two rehabilitation protocols, we found no significant differences between the groups (face-to-face and home) regarding the outcomes analyzed (pain, kinesiophobia, functional capacity, ROM and muscle strength). To our knowledge, this was the first randomized controlled and blinded clinical trial to compare two distinct forms of rehabilitation-a supervised face-to-face exercise protocol performed in the clinical setting, and a home-based exercise program with remote monitoring-in patients undergoing THA surgery. 

Both showed improved functional capacity after physical therapy follow-up. Galea et al. ^(^ ^10^ findings corroborate ours, showing no differences between the groups in self-reported functional capacity. In their study, however, the supervised face-to-face exercise group performed significantly better on the TUG test. ^(^ ^10^ ^)^ A population-based study showed that differences greater than 2.4 seconds on the TUG test can be considered clinically relevant. ^(^ ^18^ We can therefore consider that even without statistically significant differences, there was a clinically relevant improvement in the objectively measured functional capacity in both groups after the interventions, since the individuals showed reductions between 7.80 and 26.90 seconds on the TUG test. ^(^ ^18^ Notably, in the study by Galea et al. ^(^ ^10^, the home group did not receive calls or follow-up during the intervention period, and the evaluators were not blinded to the participant allocation. 

Both protocols were able to increase the torque peaks of the operated lower limb’s abductors and external rotators. Even though FG used shin guards, we observed no significant differences in the torque peaks between groups, suggesting that in this phase of rehabilitation the use of an external load increment seems not to add benefits to the treatment. Considering that evidence points to hip abductor weakness as one of the main deficits found in THA patients^16^ ^),(^ ^17^, and given its fundamental role in maintaining posture stability, trunk control and gait performance, rehabilitation protocols that facilitate its rapid recovery are of paramount importance. ^(^ ^19^ Contrary to our results, Unlu et al. ^(^ ^4^ observed higher hip muscle strength values in the group that performed supervised face-to-face exercises. However, the groups showed a significant difference in torque peaks in the pre-intervention evaluation, which may have influenced the study results. ^(^ ^4^

No studies with a methodology similar to ours that evaluated the ROM of THA patients were found in the literature. Although punctual improvements were observed in ROM, from a clinical perspective both groups persist with important limitations. According to Polkowski et al. ^(^ ^20^, a typical individual needs between 67-124 degrees of flexion, 18-33 degrees of abduction, and 15-26 degrees of external hip rotation to perform functional activities like tying shoes, going up and down stairs, sit down and get up from a chair. 

Study limitations include the absence of a longer follow-up period and a possible response bias in relation to the exercising records of the TG participants, since this information was self-reported. 

Given the difficulties in accessing traditional physical therapy treatment, the results of the present study, in agreement with previous studies on home-based treatment and/or tele-rehabilitation in patients with lower limb arthroplasty^7^ ^),(^ ^10^ ^),(^ ^15^, point to home exercises associated with remote monitoring as an alternative in the physical therapy treatment of THA patients.

## CONCLUSION

Our findings indicate no differences between a supervised face-to-face exercise protocol and a tele-rehabilitation program among patients in the recent THA postoperative period for the outcomes of pain, kinesiophobia, functional capacity, ROM and muscle strength. Both forms of rehabilitation were able to improve the functional capacity, range of motion and muscle strength in postoperative individuals, and proved to be safe and easy to reproduce.
